# Identification of KPC-112 from an ST15 Klebsiella pneumoniae Strain Conferring Resistance to Ceftazidime-Avibactam

**DOI:** 10.1128/msphere.00487-22

**Published:** 2022-11-14

**Authors:** Siquan Shen, Chengkang Tang, Li Ding, Renru Han, Dandan Yin, Weiwei Yang, Yan Guo, Fupin Hu

**Affiliations:** a Institute of Antibiotics, Huashan Hospital, Fudan University, Shanghai, China; b Key Laboratory of Clinical Pharmacology of Antibiotics, Ministry of Health, Shanghai, China; Antimicrobial Development Specialists, LLC

**Keywords:** carbapenem-resistant *Enterobacterales*, ceftazidime-avibactam, *Klebsiella pneumoniae*, KPC-112

## Abstract

Ceftazidime-avibactam is an effective antibiotic combination of a β-lactam and a β-lactamase inhibitor against Klebsiella pneumoniae-carbapenemase (KPC)-producing *Enterobacterales*. Despite a relatively low resistance rate, reports of resistance to ceftazidime-avibactam mainly caused by the mutations in KPC have increased in recent years. Here, we report a ceftazidime-avibactam-resistant and carbapenem-susceptible Klebsiella pneumoniae strain carrying a novel KPC variant, KPC-112, which differs from KPC-2 by 4-amino-acid deletions at Ambler positions 166L/167E and 242G/243T. The isolate was identified as K. pneumoniae by a Vitek mass spectrometer (bioMérieux, France). The MICs of antimicrobial agents were determined using broth microdilution susceptibility method. The result showed that the isolate was resistant to ceftazidime-avibactam (MIC = >128 mg/L) but susceptible to imipenem (MIC = 0.5 mg/L), meropenem (MIC = 1 mg/L), and tigecycline (MIC = 2 mg/L). The carbapenemase genes were confirmed by PCR-based sequencing. Plasmid transformation assay showed that the *bla*_KPC-112_-positive transformant increased MICs of ceftazidime-avibactam, ceftazidime, and cefepime by at least 256-fold, 128-fold, and 128-fold, respectively, compared with the recipient Escherichia coli DH5α. According to the whole-genome sequencing analysis, many common resistance genes were identified, including *bla*_KPC-112_, *bla*_OXA-1_, *bla*_CTX-M-15_, *bla*_TEM-1B_, *bla*_SHV-28_, *aac(6′)Ib-cr*, *aac(3)-IId*, *qnrS1*, *catA2*, *catB4*, and *fosA6*, and mutations of GyrA (GyrA-83F and GyrA-87A) and ParC (ParC-80I) were also found. Overall, our study highlights the importance of monitoring susceptibility during ceftazidime-avibactam treatment and accurate detection of KPC variants.

**IMPORTANCE** Carbapenem-resistant *Enterobacterales* (CRE) are one of the most serious antimicrobial resistance problems in the world, listed as an “urgent” threat by the U.S. Centers for Disease Control and Prevention. Among CRE, K. pneumoniae-carbapenemase-producing Klebsiella pneumoniae (KPC-KP) has become a significant health threat due to its rapid transmissibility and high mortality. With the wider clinical use of ceftazidime-avibactam, reports of resistance have increased in recent years even though the overall resistance rate remains relatively low. Among the reported resistance mechanisms are mainly mutations derived from the *bla*_KPC-2_ or *bla*_KPC-3_ gene. Here, we describe the characterization of a ceftazidime-avibactam-resistant *bla*_KPC-112_-positive K. pneumoniae clinical isolate for the first time. A number of *Enterobacteriaceae* isolates producing these kinds of KPC variants might be missed by conventional antimicrobial susceptibility testing (AST) methods and lead to irrational drug use. So, this study of KPC-112 will help to establish the diversity of KPCs and remind researchers of the challenge of drug resistance and detection brought by the KPC variants.

## INTRODUCTION

Carbapenem-resistant *Enterobacterales* (CRE) are one of the most serious antimicrobial resistance problems in the world, listed as an “urgent” threat by the U.S. Centers for Disease Control and Prevention (1). Among CRE, Klebsiella pneumoniae-carbapenemase-producing Klebsiella pneumoniae (KPC-KP) has become a significant health threat due to its rapid transmissibility and high mortality (2). KPC, an Ambler class A serine β-lactamase, can hydrolyze almost all cephalosporins and carbapenems, resulting in the lack of effective treatment options (3). As a result, tigecycline and polymyxin B have served as the last resort for multidrug-resistant Gram-negative bacteria (4). However, their clinical application has been limited by toxicity, induced resistance, and high economic burden (5).

Given the high-level resistance caused by KPC-KP, a novel combination of β-lactam and β-lactamase inhibitor, ceftazidime-avibactam (CZA), was approved in China in 2019 with potent activity against KPC/OXA-48-like-producing bacteria (6). However, with the wider clinical use, reports of resistance to ceftazidime-avibactam have increased in recent years even though the overall resistance rate remains relatively low (7). Among the reported resistance mechanisms, mutations derived from the *bla*_KPC-2_ or *bla*_KPC-3_ gene are the main reasons and often contribute to high-level resistance and treatment failure subsequently (8). More importantly, the number of newly identified KPC variants within the last 2 years has exceeded that of the past 2 decades. To date, more than 130 *bla*_KPC_ subtypes have been reported in the world according to the NCBI database. Most of the novel *bla*_KPC_ variants reported in China were mutated from *bla*_KPC-2_, while those in Europe were mainly from *bla*_KPC-3_ (9, 10).

In this study, we describe the characterization of *bla*_KPC-112_, a novel *bla*_KPC_ variant that confers resistance to ceftazidime-avibactam and restored susceptibility to carbapenems.

## RESULTS

### Overview of the K. pneumoniae clinical isolate.

The isolate K. pneumoniae HS5432 was collected in a sputum sample from a 92-year-old male patient admitted to Huashan Hospital, Fudan University, in 2021. The patient had been hospitalized for several months after suffering a cerebral ischemic stroke. During this time, the patient had a recurrent respiratory infection as well as a urinary tract infection accompanied by the detection of carbapenem-resistant K. pneumoniae. Empirical therapy included cefmetazole (2.0 g every 12 h for 6 days) plus doxycycline (0.1 g every 12 h for 3 days), and cefoperazone-sulbactam (3.0 g every 12 h for 6 days) had no effect. Considering the poor therapeutic response, ceftazidime-avibactam (2.5 g every 12 h) was administered against the infection. Fortunately, the patient recovered after ~2 weeks of treatment with ceftazidime-avibactam. Nevertheless, 1.5 months after treatment was discontinued, urinary tract infection recrudesced, and one ceftazidime-avibactam-resistant K. pneumoniae isolate, HS5432, was isolated from sputum. The antimicrobial susceptibility profiles of K. pneumoniae HS5432 are presented in Table 1. They showed that K. pneumoniae HS5432 was resistant to ceftazidime (MIC = >32 mg/L), cefepime (MIC = >128 mg/L), ciprofloxacin (MIC = >8 mg/L), piperacillin-tazobactam (MIC = >256 mg/L), trimethoprim-sulfamethoxazole (MIC = >32 mg/L), and ceftazidime-avibactam (MIC = >128 mg/L) but sensitive to imipenem (MIC = 0.5 mg/L), meropenem (MIC = 1 mg/L), and tigecycline (MIC = 2 mg/L).

**TABLE 1:** Susceptibility of *bla*_KPC-112_-positive K. pneumoniae clinical isolate 5432, transformant, and recipient to antimicrobial agents

Strain	Carbapenemase gene	MIC (mg/L) of agent^*a*^									
		CZA	IPM	MEM	FEP	CAZ	TZP	AMK	SXT	CIP	TGC
K. pneumoniae HS5432	*bla*_KPC-112_	>128	0.5	1	>128	>32	>256	>128	>32	>8	2
E. coli DH5α-HS5432-T	*bla*_KPC-112_	64	0.12	≤0.03	8	>32	≤2	≤1	≤0.25	≤0.06	0.12
E. coli DH5α		≤0.125	≤0.06	≤0.03	≤0.06	≤0.25	≤2	≤1	≤0.25	≤0.06	≤0.06

a Abbreviations: CZA, ceftazidime-avibactam; IPM, imipenem; MEM, meropenem; FEP, cefepime; CAZ, ceftazidime; TZP, piperacillin-tazobactam; AMK, amikacin; SXT, trimethoprim-sulfamethoxazole; CIP, ciprofloxacin; TGC, tigecycline.

### Carbapenemase and plasmid transformation assay.

PCR and Sanger sequencing of the full-length *bla*_KPC_ gene revealed a novel *bla*_KPC_ variant, designated *bla*_KPC-112_. Nucleotide alignment of different *bla*_KPC_ variants showed that *bla*_KPC-112_ differs from *bla*_KPC-2_ by two 6-bp deletions (nucleotide positions 492 to 497 and positions 721 to 726), resulting in 4-amino-acid deletions at Ambler positions 166E/167L and 242G/243T. According to the results of the plasmid transformation assay, the transformant was positive for *bla*_KPC-112_ and increased MICs of ceftazidime-avibactam, ceftazidime, and cefepime by at least 256-fold, 128-fold, and 128-fold, respectively, compared with the recipient Escherichia coli DH5α (Table 1).

### WGS analysis and characterization of *bla*_KPC-112_-carrying plasmid.

**FIG 1:**
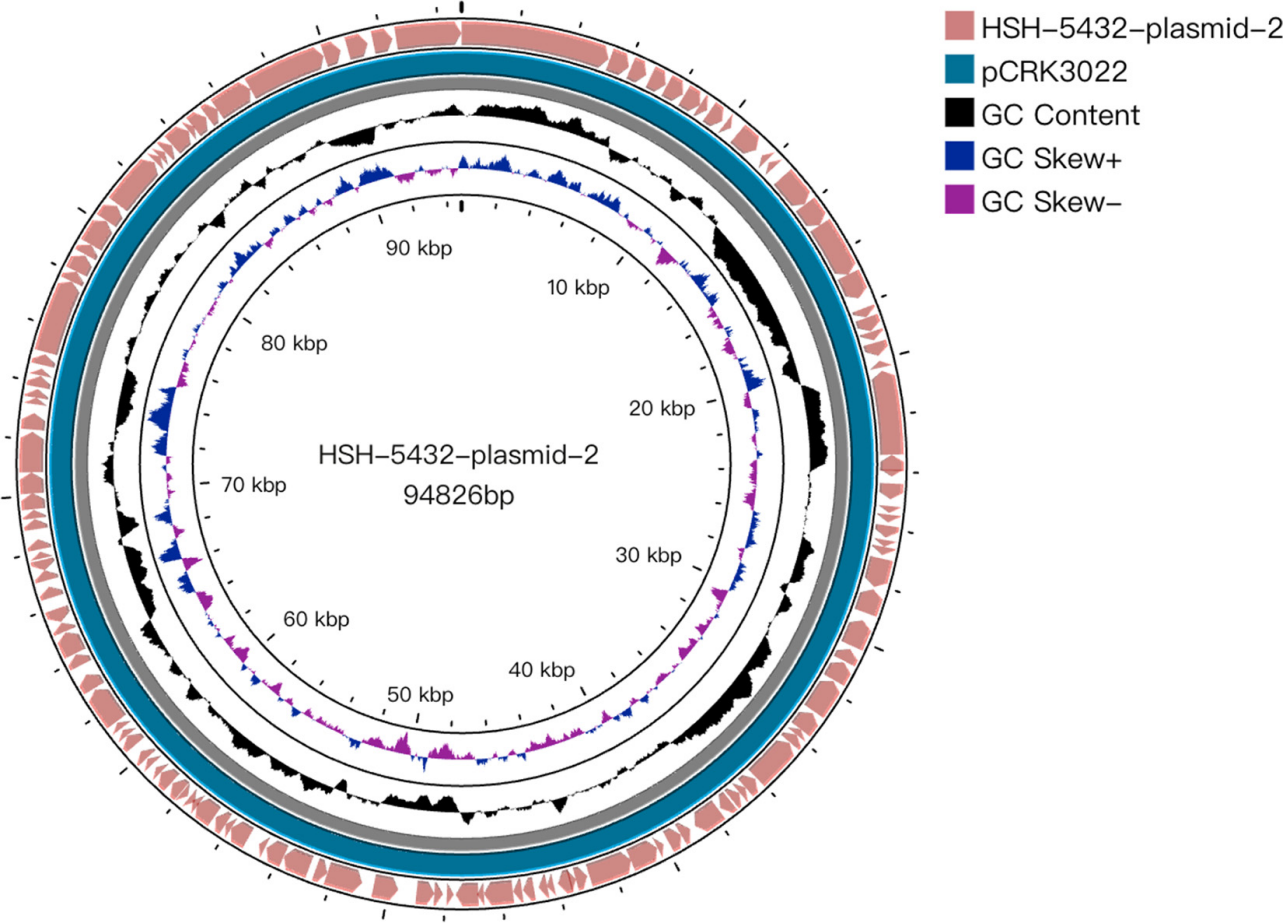
Alignments of plasmids. Comparison of the plasmids HSH-5432-plasmid-2 and pCRK3022-2 using Proksee. A BLAST search for the sequence in GenBank showed that the sequence of HSH-5432-plasmid-2 was very similar (99.95% coverage and 100% identity) to that of pCRK3022-2 (123,202 bp, GenBank accession no. CP091332), a plasmid of K. pneumoniae isolated from Jiangsu, China.

**FIG 2:**
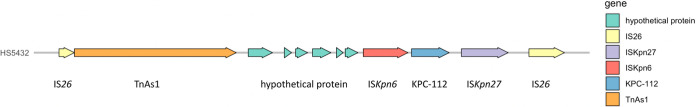
The genetic environment surrounding *bla*_KPC-112_. Genes, genetic elements, and other traits are color coded according to functional classification.

According to the whole-genome sequencing (WGS) analysis, many resistance genes had been identified, including the β-lactamase genes *bla*_KPC-112_, *bla*_OXA-1_, *bla*_CTX-M-15_, *bla*_TEM-1B_, and *bla*_SHV-28_, the aminoglycoside resistance genes *aac(6′)Ib-cr* and *aac(3)-IId*, the fluoroquinolone resistance gene *qnrS1*, the phenicol resistance genes *catA2* and *catB4*, and the fosfomycin resistance gene *fosA6*. Quinolone-related resistance gene mutations of GyrA (GyrA-83F and GyrA-87A) and ParC (ParC-80I) were found in K. pneumoniae HS5432. According to the multilocus sequence typing result, strain HS5432 belonged to ST15. The *bla*_KPC-112_ gene was carried by a 94,826-bp plasmid, HSH-5432-plasmid-2, there was no known replicon predicted in plasmid HSH-5432-plasmid-2 by PlasmidFinder, and no other resistance gene was found in this plasmid. A Basic Local Alignment Search Tool (BLAST) search of the sequence in GenBank showed that the sequence of HSH-5432-plasmid-2 was very similar (99.95% coverage and 100% identity) to that of pCRK3022-2 (123,020 bp, GenBank accession no. CP091332), a plasmid of K. pneumoniae isolated from Nantong, China (Fig. 1). The genetic structure of *bla*_KPC-112_ in HSH-5432-plasmid-2 is identical to pCRK3022-2, carrying an IS*26*-based composite transposon, which is an 11.6-kb region including *bla*_KPC-2_ and two flanking IS*26* elements; the complete genetic structure was IS*26*-*tnpR*-IS*Kpn27*-*bla*_KPC-2_-IS*Kpn6*-IS*26* (Fig. 2).

## DISCUSSION

With the global dissemination of KPC-producing K. pneumoniae (KPC-KP), ceftazidime-avibactam has become one of the few effective treatment alternatives against KPC-KP since its approval for application in clinics (11). However, acquired resistance has been increasingly reported in multiple independent occurrences (12). The resistance may be due to (i) coproduction of metallo-β-lactamases (13), (ii) overexpression of *bla*_KPC_ along with outer membrane porin loss (14), or (iii) mutations derived from the *bla*_KPC-2_ or *bla*_KPC-3_ gene, which is the major molecular mechanism. To the best of our knowledge, amino acid substitutions, mainly D179Y, are the most frequent mutation, followed by amino acid insertions and deletions (15, 16).

In this study, we identified, sequenced, and characterized a KPC-112-producing ST15-type K. pneumoniae clinical strain conferring resistance to ceftazidime-avibactam. The exposure to ceftazidime-avibactam likely contributed to its selection *in vivo* (17). Compared with KPC-2, KPC-112 has 4-amino-acid deletions at Ambler positions 166E/167L and 242G/243T. It is worth noting that mutations within the Ω-loop (Arg_164_ to Asp_179_) that embrace the active site of KPC have been proven to enhance ceftazidime affinity (18) and restrict avibactam binding (19). So far, it is reported that many mutants with mutations in the Ω loop, such as KPC-12, KPC-31, KPC-33, KPC-35, KPC-39, and KPC-57, may mediate ceftazidime-avibactam resistance via the mutations followed by D179Y (9), L169M (20), L169P (20), A172T (21), and D179V (14). In addition, mutations within another loop (Cys_238_ to Thr_243_) seem to exhibit a weaker effect than those within the Ω loop. The impact of KPC-3 variants on the increase of ceftazidime-avibactam MICs was as follows: D179Y/T243M > D179Y > V240G (22).

Two major genetic structures, the Tn*4401* transposon and the Tn*3-*Tn*4401* transposon chimera are mostly associated with KPC-2 (23–25). The Tn*4401* transposon, with a length of 10 kb, is considered the original genetic structure mediating *bla*_KPC_ gene acquisition worldwide, and a total of eight unique isoforms (a to h) differing by deletions immediately upstream of the *bla*_KPC_ gene have been characterized, with Tn*4401a* and Tn*4401b* being the most common (23, 26–28). Tn*4401* is composed of two insertion sequences, IS*Kpn6* and IS*Kpn7*, a transposase gene (*tnpA*), a resolvase gene (t*npR*), and *bla*_KPC_ (25). In China, the genetic environment of the *bla*_KPC_ gene was distinct, with the Tn*3*-Tn*4401* transposon chimera being the most common (29, 30). The full gene order of the Tn*3*-Tn*4401* transposon chimera was Tn*3*-*tnpA*, Tn*3*-*tnpR*, IS*Kpn8*, *bla*_KPC_, IS*Kpn6*-like element, Tn*1721* resolvase, and Tn*1721* transposase (24, 29, 30). However, the genetic structure of *bla*_KPC-112_ in our study was different; *bla*_KPC-112_ was carried by an 11.6-kb region, with two flanking IS*26* elements that resembled a composite transposon. It is known that two copies of IS*26* can form a composite transposon, which can be excised from the plasmid to form a translocation unit (TU) (31–33). Combined with previous studies, we speculate that the presence of IS*26*-mediated compound transposons may be more conducive to the spread of KPC genes, a possibility that requires extensive attention (34).

As mentioned above, the KPC-2/KPC-3 variants seem to exhibit decreasing carbapenemase activity, raising a challenge for routine carbapenemase detection assays (35). KPC variants usually show false-negative results toward methods including carbapenemase inhibitor enhancement testing, the modified carbapenem inactivation method (mCIM)/EDTA-modified CIM (eCIM) recommended by the CLSI, and the NG-Test Carba 5 (35). The misleading detection in the clinical laboratory may lead to incorrect clinical decision-making and treatment failure. Therefore, to improve the specificity of detection, the phenotype assay, as well as molecular testing, should be carried out to detect all KPC subtypes.

In conclusion, it is important for clinicians to monitor the susceptibility of KPC-producing strains to ceftazidime-avibactam and detect KPC variants at the early stage during therapy. A more accurate and detailed characterization should be provided to help adjust the dosage of ceftazidime-avibactam and therapeutic regimens promptly, thus preventing therapy failure and containing the wide spread of ceftazidime-avibactam-resistant strains.

## MATERIALS AND METHODS

### Species identification, AST, and confirmation of carbapenemase production.

Strain identification was performed by a Vitek mass spectrometer (bioMérieux, France). The MIC of antimicrobials was determined using broth microdilution susceptibility testing. E. coli ATCC 25922 was used as quality control for antimicrobial susceptibility testing (AST). Quality control and MIC results were interpreted according to the CLSI breakpoints for all agents except tigecycline, which was interpreted according to the guidelines of the Food and Drug Administration. Carbapenemase production was phenotypically detected using APB/EDTA (carbapenemase inhibitor APB [3-aminophenylboronic acid] and EDTA enhancement method). *bla*_KPC_ was confirmed by PCR-based sequencing (36).

### Plasmid transformation assay.

Plasmid transformation assays were carried out to explore the characteristics of the *bla*_KPC-112_-carrying plasmids using E. coli DH5α as a recipient strain. The transformants were selected on Mueller-Hinton agar supplemented with ceftazidime-avibactam (16 μg/mL). The presence of *bla*_KPC-112_ was confirmed by PCR and PCR-based sequencing. The presence of resistance genes in the transformant was confirmed by PCR and PCR-based sequencing.

### Whole-genome sequencing and bioinformatics analysis.

The isolates’ genomic DNA was obtained by using a Qiagen commercial kit according to the manufacturer’s recommendation. The genomic DNA was sequenced by using an Illumina MiSeq platform (Illumina Inc.) with a paired-end approach (2 by 300 bp). SPAdes 3.12.0 was used to *de novo* assemble the sequencing reads. Analysis of antimicrobial resistance genes as well as multilocus sequence typing (MLST), detection of mobile elements and resistance-related mutants, open reading frame prediction, and annotation was done with ResFinder (37), BIGSdb-Pasteur MLST (38), ISfinder (39), RAST version 2.0 (40) (https://rast.nmpdr.org), and BLAST (41) (https://blast.ncbi.nlm.nih.gov/Blast.cgi). The genetic environment surrounding the target gene was analyzed by Easyfig tools (42) (http://mjsull.github.io/Easyfig) and Proksee (https://proksee.ca/). The conjugation elements were detected by using oriTfinder, a web-based tool for the identification of the origin of transfers in DNA sequences of bacterial mobile genetic elements (43).

The study protocol was approved by the Institutional Review Board of Huashan Hospital, Fudan University (no. 2018-408).

### Data availability.

The genome sequencing data are publicly available at NCBI GenBank under the BioProject accession number CP107245.
